# Nodal radiotherapy for prostate adenocarcinoma recurrence: predictive factors for efficacy

**DOI:** 10.3389/fonc.2024.1468248

**Published:** 2024-10-25

**Authors:** Anna Gueiderikh, Jérémy Baude, David Baron, Renaud Schiappa, Sandrine Katsahian, Damien Moreau, Marc Laurans, Jean-Emmanuel Bibault, Sarah Kreps, Pierre-Yves Bondiau, Magali Quivrin, Alexis Lépinoy, David Pasquier, Jean-Michel Hannoun-Levi, Philippe Giraud

**Affiliations:** ^1^ Radiation Oncology Department, Georges Pompidou European Hospital, Assistance Publique – Hôpitaux de Paris, Paris Descartes University, Paris Sorbonne Cité, Paris, France; ^2^ Radiation Oncology Department, Centre Antoine Lacassagne, Nice, France; ^3^ Radiation Oncology Department, Centre Georges-François Leclerc, Dijon, France; ^4^ Epidemiology, Biostatistic and Health Data Department, University Cote d’Azur, Centre Antoine Lacassagne, Nice, France; ^5^ Université Paris Cité, Paris, France; ^6^ AP-HP, hôpital européen Georges-Pompidou, Unité de Recherche Clinique, Assistance Publique – Hôpitaux de Paris (APHP) Centre, Paris, France; ^7^ Institut National de la Santé et de la Recherche Médicale (INSERM), Centre d’Investigation Clinique 1418 (CIC1418) Épidémiologie Clinique, Paris, France; ^8^ Institut National de la Santé et de la Recherche Médicale (INSERM) UMR_S 1138 équipe 22, Centre de Recherche des Cordeliers, Paris, France; ^9^ Radiation Oncology Department, Institut de Cancérologie de Bourgogne, Dijon, France; ^10^ Academic Department of Radiation Oncology, Centre O. Lambret, Lille, France; ^11^ Univ. Lille, CNRS, Centrale Lille, UMR 9189 - CRIStAL, Lille, France

**Keywords:** nodal recurrence, prostate adenocarcinoma, whole pelvic radiation therapy, SBRT (stereotactic body radiation therapy), androgen deprivation therapy (ADT), PSA doubling time

## Abstract

**Background:**

Nodes are the second site for prostate cancer recurrence. Whole-pelvic radiotherapy (WPRT) has shown superiority over nodal stereotactic body radiotherapy (SBRT) in two retrospective cohorts. We aimed to compare both modalities and assess factors associated with treatment outcomes.

**Materials and methods:**

This retrospective multicentric cohort study included patients from five institutions spanning from 2010 to 2022. Patients had a history of prostatic adenocarcinoma classified as N0 M0 at diagnosis with a first nodal-only pelvic castration-sensitive recurrence. Failure-free survival (FFS) was defined as the time from the end of RT to the first failure event–biochemical or imaging recurrence, or death.

**Results:**

A total of 147 patients (pts) were analyzed, mainly treated for a recurrence after initial prostatectomy (87%), with 64 (43.5%) undergoing SBRT and 83 (56.5%) undergoing WPRT. SBRT was chosen mainly for dosimetric constraints (67%) and was associated with a lower rate of concomitant androgen deprivation therapy (ADT) prescription. With a median follow-up of 68 months [inter-quartile range (IQR) = 51], FFS was significantly lower in the SBRT group (p < 0.0001). In multivariable analysis, WPRT and ADT were associated with a longer FFS. Factors associated with a longer FFS after SBRT included associated ADT, lower prostate-specific antigen (PSA) levels, a PSA doubling time >6 months, and a Gleason score <8. SBRT was associated with a lower rate of genitourinary and gastrointestinal grade ≥2 complications.

**Discussion:**

For an isolated pelvic nodal prostate cancer recurrence, SBRT is associated with a shorter FFS compared to WPRT. SBRT is often more convenient for patients and leaves further pelvic salvage options available, so it can be explored as an option for well-informed patients.

## Introduction

Biochemical recurrence following radical treatment for prostate cancer occurs in one-fifth to one-half of patients 5 to 15 years after prostatectomy (1–3). Of those biochemical recurrences, 30% to 90% are identified as metastatic by metabolic imaging, depending on the tracer used (4), and lymph nodes (LNs) are the second recurrence site after bone (5). As nodal recurrence often does not shorten overall survival (6), different therapeutic strategies can be proposed. Therapeutic approaches, including radiotherapy (RT) with or without androgen deprivation therapy (ADT), vary depending on factors such as previous pelvic RT, indicated in intermediate with a high Roach score (7–10) and high-risk prostate cancer patients (11–13). It can be delivered through stereotactic body radiotherapy (SBRT) targeting only the metastatic nodes or through a large pelvic field by intensity-modulated radiotherapy (IMRT) with, often, a boost delivered to the metastatic (hypermetabolic) node (using either IMRT or SBRT).

Patients not formerly irradiated on pelvic LN (PLN) are candidates for both irradiation techniques. In this population, head-to-head comparison of whole-pelvic RT (WPRT) and SBRT has shown the superiority of WPRT in terms of recurrence-free survival in retrospective cohorts (14, 15). However, SBRT offers the advantage of a quick treatment with few side effects and is still considered (16) for recurrences not exceeding three to five nodes, sometimes repeated in the same patient (16, 17). The PEACE V-STORM randomized phase II trial is currently comparing both techniques prospectively and has already shown a similar tolerance profile of WPRT compared to SBRT (18, 19). The superiority of WPRT in terms of biochemical and regional relapse-free survival has been reported at the European Society for Radiotherapy and Oncology (ESTRO) 2024 congress, and data about metastasis-free survival are being awaited.

Several factors were associated with encouraging results in terms of recurrence after prostatectomy in large multicentric studies: Gleason score <8–10 or corresponding International Society of Urological Pathology (ISUP) group, prostate-specific antigen (PSA) doubling time >9–18 months, or an interval from primary RT to biochemical failure (IBF) ranging from 12 months to 36 months (3, 20, 21). Some other factors associated with SBRT efficacy were proposed in monocentric studies: long PSA doubling time (17) or one single involved node (22). Long IBF was identified for nodal RT efficacy independently of the treatment technique (15).

The adjunction of ADT in some but not all patients is a strong confounding factor in all studies. While at the time of initial disease, ADT increases distant metastasis-free survival and overall survival when associated with RT (23), in case of recurrence, ADT decreases the risk of biochemical failure, but it is not associated with improved survival (24–26). Phase II studies reported encouraging oncological outcomes in the case of a combination of ADT and WPRT for nodal recurrence (27), but no randomized trial demonstrated the benefit of this combination for PLN treatment. Practices are thus variable regarding ADT prescription for nodal recurrence.

In this multicentric retrospective cohort, we intended to reassess the place of SBRT in nodal recurrence treatment compared to WPRT and identify predictive factors for treatment outcome.

## Materials and methods

### Patients and treatment

Our retrospective cohort included patients from five French institutions treated by nodal RT between 2010 and 2022. They presented a first hormone-sensitive PLN recurrence (maximum of five pathological nodes) after the treatment of a primary N0 M0 prostate cancer. The treatment consisted of either SBRT to the involved LN or WPRT-pelvic IMRT with a boost delivered to the involved nodes (IMRT or SBRT).

Prior treatment of a biochemical recurrence with prostatic bed RT was accepted. Concomitant treatment of prostatic bed RT was also accepted if no target was visualized inside the prostate at the time of recurrence. Concurrent ADT was accepted. Patients were excluded if they had lymph node dissection prior to RT at recurrence, lumboaortic nodal recurrence, partial pelvic volumes RT (i.e., hemi-pelvic large field RT), concomitant non-nodal metastasis at recurrence, and treatment delivered partially in different treatment centers. Some patients from three of the five centers were part of previously published cohorts (14, 15, 27, 28).

LN topology was described according to anatomical drainage; obturator and inguinal LNs were grouped with iliac external LN, and sacral LNs were grouped with iliac internal LN. Iliac common LNs were analyzed separately. The higher LN group treated was reported.

The study was carried out in accordance with the French ethic MR004 guidelines, in compliance with the Declaration of Helsinki, and approved by the CER APHP Centre ethics committee (No. IRB: IORG0010044). Patients were informed about the use of their data, and those who declined access to their medical files were excluded from the study.

### Outcomes

The primary objective was to compare the outcomes of nodal SBRT and WPRT + boost in terms of failure-free survival (FFS). Secondary objectives were to compare SBRT and WPRT in terms of overall survival (OS), to compare nodal SBRT and IMRT boost to the involved nodes during WPRT, to describe the reasons for an SBRT treatment choice, and to identify predictive factors for treatment outcomes.

Biochemical recurrence was defined as PSA > 2 ng/mL or a value superior to the pre-treatment PSA. Local progression and distant progression were defined in next-generation medical imaging. Local progression was defined as a progression in the same nodal area as the one treated. Distant progression was defined as a progression in another nodal area or metastatic progression. OS and progression-free survival (PFS) were calculated from the end of radiotherapy and estimated using the Kaplan–Meier method. FFS was calculated from the end of radiotherapy to the first event between biochemical recurrence, progression, and death. Patients without an event at the time of the last follow-up were censored at this date.

Toxicity outcomes were graded retrospectively according to the Common Terminology Criteria for Adverse Events (CTCAE) v4.0 classification.

### Statistical analysis

The median follow-up and associated interquartile range (IQR) were estimated using the reversed Kaplan–Meier method. SBRT- and IMRT-treated groups were compared using the Wilcoxon test for continuous characteristics, and a chi-squared or Fisher’s test was used to compare the proportions of categorical variables. Statistical analysis did not include missing data. A multivariable Cox regression model was used to assess the factors associated with FFS. Models were compared using the Akaike information criterion (AIC) and the Bayesian information criterion (BIC).

All tests were two-sided. A p-value ≤0.05 indicated statistically significant factors.

The R software (version 4.2.2) was used for all statistical analyses.

## Results

Our cohort was made of 147 patients. Sixty-four (43.5%) and 83 patients (56.5%) underwent SBRT and WPRT, respectively [including boosts: 24 (29%) SBRT and 59 (71%) IMRT]. No significant difference was observed between the two treatment groups regarding age at the time of primary as well as recurrence treatment, with homogeneous patient distribution among the participating centers. LNs were imaged by PET choline in 90% of patients (**Supplementary Table S1**). Regarding initial prostate tumor characteristics, the Gleason stage at diagnosis was similar between groups, but the SBRT group presented a trend for a lower PSA and higher T2b score at diagnosis (**Table 1**). According to the inclusion criteria, all patients were N0 M0 at diagnosis. Patients undergoing SBRT were treated for a lower number of involved LN at recurrence (in the SBRT group, 81% of patients were treated for one LN vs. 57% of patients in the WPRT group, p < 0.01, **Table 1**). SBRT was never chosen when iliac external and iliac internal territories were simultaneously affected. Patients treated with SBRT tended to present a higher IBF and lower PSA value at recurrence (**Table 1**). PSA values at recurrence were also analyzed according to the initially received treatment and reported in **Supplementary Table S2**.

**Table 1:** Population characteristics.

	Total (N = 147)	Nodal SBRT (N = 64)	WPRT + Boost (N = 83)	p-Value
Gleason score at diagnosis				0.60
- 6 and less	30 (22%)	16 (27%)	14 (18%)	(1)
- 7 (3 + 4)	48 (35%)	21 (35%)	27 (35%)	
- 7 (4 + 3)	33 (24%)	13 (22%)	20 (26%)	
- 8 and more	27 (20%)	10 (17%)	17 (22%)	
- Missing	9	4	5	
T score at diagnosis				0.06
- 2a and less	16 (11%)	3 (5%)	13 (16%)	(3)
- 2b	23 (16%)	13 (22%)	10 (12%)	
- 2c and more	102 (72%)	44 (73%)	58 (72%)	
- Missing	6	4	2	
PSA value at diagnosis (ng/mL)				0.10
- Median (range)	8.3 (1.4, 72.3)	7.7 (1.4, 72.3)	8.8 (2.5, 64.0)	(2)
- Missing	30	15	15	
Number of nodes treated				<0.01
- 1	99 (67%)	52 (81%)	47 (57%)	(1)
- 2	34 (23%)	11 (17%)	23 (28%)	
- 3 to 5	14 (10%)	1 (2%)	13 (16%)	
Topography of involved LN				0.10
- Iliac common	26 (18%)	10 (16%)	16 (19%)	(3)
- Iliac external	72 (49%)	36 (56%)	36 (43%)	
- Iliac internal	39 (27%)	18 (28%)	21 (25%)	
- Iliac internal + Iliac external	10 (7%)	0 (0%)	10 (12%)	
Interval to biochemical failure (IBF) (years)				0.16
- Median (range)	6.8 (0.0, 18.5)	8.0 (1.3, 17.2)	5.9 (0.0, 18.5)	(2)
PSA value at nodal recurrence radiotherapy (ng/mL)				0.06
- Median (range)	2.0 (0.1, 20.0)	1.8 (0.1, 13.8)	2.3 (0.3, 20.0)	(2)

1) Pearson’s chi-squared test; 2) Wilcoxon rank sum test; 3) Fisher’s exact test for count data. SBRT, stereotactic body radiotherapy; WPRT, whole-pelvic radiotherapy; PSA, prostate serum antigen.

Initial disease treatment was mainly prostatectomy for both groups (n = 127, 87%). Rates of PLN dissection and associated ADT for primitive treatment were similar between groups (**Table 2**). SBRT-treated patients had more often a prior prostate bed irradiation for a biochemical recurrence (73% of SBRT-treated patients had a pelvic loge RT against 48% of IMRT-treated patients, p < 0.01, **Table 2**), and at recurrence, 32% of the WPRT-treated patients presented a concomitant prostate bed irradiation. Accordingly, reasons for SBRT delivery at recurrence, when indicated in the medical files, were mainly dosimetric analysis (n = 18, 67%), followed by patient’s choice (n = 4, 15%), toxicity of former RT (n = 3, 11%), or patient’s medical history advising for especial care for organs at risk (n = 2, 7%) (**Supplementary Table S3**).

**Table 2:** Treatment characteristics.

	Total (N = 147)	Nodal SBRT (N = 64)	WPRT + Boost (N = 83)	p-Value
Initial treatment:				
Radical prostatectomy				0.87 (1)
- No	19 (13%)	8 (12%)	11 (13%)	
- Yes	127 (87%)	56 (88%)	71 (87%)	
- Missing	1	0	1	
Pelvic lymph node dissection				0.39 (1)
- No	51 (39%)	24 (43%)	27 (36%)	
- Yes	81 (61%)	32 (57%)	49 (64%)	
- Missing	15	8	7	
RT to the prostate	17 (12%)	7 (11%)	10 (12%)	0.83 (1)
Associated ADT				0.27 (1)
- No	109 (79%)	50 (83%)	59 (76%)	
- Yes	29 (21%)	10 (17%)	19 (24%)	
- Missing	9	4	5	
Adjuvant RT to the prostatic bed	87 (59%)	47 (73%)	40 (48%)	<0.01 (1)
Recurrence treatment:				
Radiotherapy dose		18 to 45 Gy in 5 to 15 Gy per fraction	50.4 Gy (45.0, 70.0) to the whole pelvis + 10–20 Gy in SIB or sequential boost	
Associated prostate bed RT			26 (32%)	
Machine used for nodal targeting (nodal SBRT or nodal boost)				
- CyberKnife	60 (41%)	41 (64%)	19 (23%)	
- Other LINAC	57 (39%)	23 (36%)	34 (42%)	
- Tomotherapy	30 (20%)	0 (0%)	30 (36%)	
Associated ADT				<0.01 (1)
- No	80 (55%)	50 (78%)	30 (37%)	
- Yes	66 (45%)	14 (22%)	52 (63%)	
- Missing	1	0	1	
**Ulterior re-irradiation of nodal areas**	35 (24%)	25 (38%)	10 (12%)	<0.01 (1)

1) Pearson’s chi-squared test. RT, radiotherapy; ADT, androgen deprivation therapy; SBRT, stereotactic body radiotherapy; WPRT, whole-pelvic radiotherapy.

At recurrence, RT delivered a median dose of 50.4 Gy to the whole pelvis with a 10 to 20 Gy boost to the pathological nodes in the WPRT group. SBRT was delivered in 18 to 45 Gy in 5 to 15 Gy per fraction. Detailed dose characteristics and the main irradiation regimen used can be found in **Supplementary Table S4**. It is to be noted that 32% of the patients who were offered WPRT had an associated prostate bed RT at the same time. SBRT was delivered with a CyberKnife™ (Accuray Corporate Headquarters, Madison, WI 53717-1954, USA) in 64% of cases and with a linear accelerator (LINAC) meeting the stereotactic criteria in 36% of cases. In the WPRT group, the nodal boost could be delivered by SBRT or by IMRT including Tomotherapy™ (Accuray Corporate Headquarters, Madison, WI 53717-1954, USA). SBRT treatment was less frequently associated with ADT than WPRT (22% of SBRT-treated patients had an associated ADT against 63% of WPRT-treated patients, p < 0.0001) (**Table 2**). ADT was initiated at failure for the other patients (data not shown).

**Figure 1:**
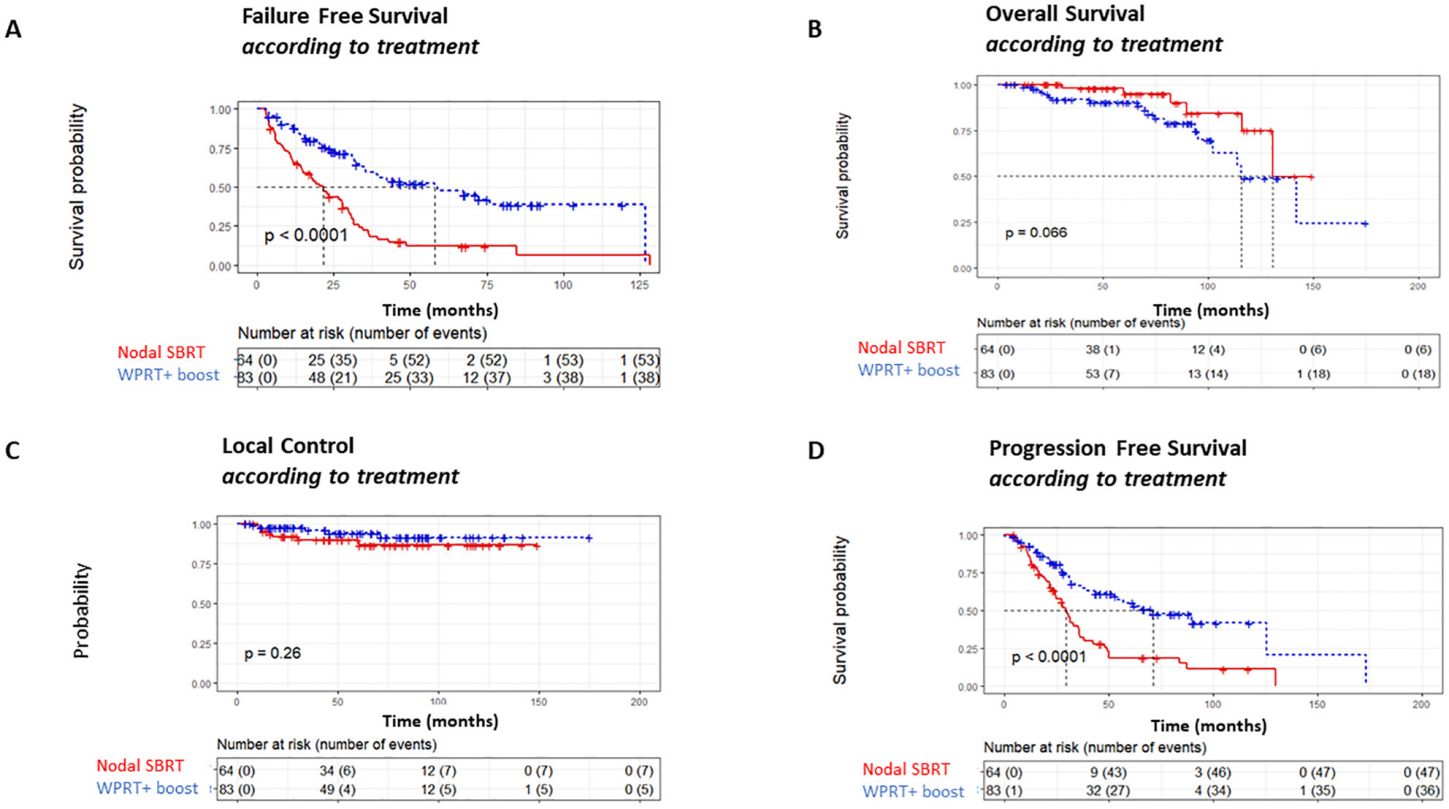
Survival data. **(A)** Failure-free survival. **(B)** Overall survival. **(C)** Local control. **(D)** Progression-free survival. SBRT, stereotactic body radiotherapy; WPRT, whole-pelvic radiotherapy.

With a median follow-up of 68 months (IQR = 51), FFS was shorter with SBRT treatment than with WPRT treatment [median FFS: 21.7 months (14.8–29.8) vs. 58.1 (35.4–NA) in the SBRT group and WPRT groups respectively, p < 0.01, **Figure 1A**]. Accordingly, the 5-year FFS was 12% (95% CI = 6%–25%) in the SBRT population against 47% (36–62%) in the WPRT-treated patients. However, this shorter FFS did not translate into a shorter OS. SBRT-treated patients had even a trend for a longer OS (**Figure 1B**). Rates of local control were similar (**Figure 1C**), and failure was thus rather a distant progression that could often be determined on imaging shortly after biochemical progression translating into a difference in PFS (**Figure 1D**). SBRT-treated patients could benefit from a second RT of the nodal area in 38% of cases, against 12% for WPRT-treated patients (p < 0.01, **Table 2**).

Toxicity data analysis revealed a lower rate of acute and late genito-urinary ≥ grade 2 as well as acute gastro-intestinal ≥ grade 2 complications after SBRT (**Supplementary Table S5**). Nine (11%) patients in the WPRT group had both acute and late urinary toxicities, and three (4%) patients had both acute and late digestive toxicities. Late complications persisted up to the end of the follow-up for 2/3 of the patients. In the WPRT group, the majority of observed acute urinary toxicities occurred in the IMRT boost group (**Supplementary Table S6**), and this modality was mainly chosen for the patients who received a concomitant prostatic bed treatment.

In the univariable model, whole-pelvic IMRT, associated ADT, and prostatic loge RT prior to treatment were associated with a longer FFS. In the multivariable analysis, only whole-pelvic RT and associated ADT remained as predictive factors for a longer FFS (**Table 3**).

Regarding the nodal boost modality, WPRT and IMRT boost were the only modalities performing better than nodal SBRT in the multivariable model (**Table 3**).

**Table 3:** Univariable and multivariable analyses of FFS (Cox survival model) in the whole population.

Variable	Univariable HR	p-Value	Multivariable HR	p-Value	Missing Obs.
**Use of ADT at recurrence**	**0.35 [0.22–0.55]**	**<0.001**	**0.40 [0.22–0.71]**	**0.002**	**1**
PSA doubling time (linear)	0.99 [0.96–1.02]	0.55			21
PSA doubling time >6 months	0.83 [0.54–1.27]	0.38			21
Time from diagnosis	1.00 [0.99–1.05]	0.85			0
PSA at diagnosis	1.01 [0.99–1.03]	0.13	1.00 [0.99–1.02]	0.68	30
PSA at recurrence	1.01 [0.96–1.06]	0.72			0
Gleason ≥8 at diagnosis	0.65 [0.36–1.17]	0.15	0.88 [0.45–1.71]	0.62	5
**Treatment modality (compared to SBRT)** **- WPRT + Nodal IMRT** **- WPRT + Nodal SBRT**	**0.33 [0.21–0.53]** **0.50 [0.25–0.98]**	**<0.001** **0.042**	**0.48 [0.27–0.86]** 0.76 [0.33–1.75]	**0.013** 0.52	**0**
Number of treated LN (compared to 1) 2 3 to 5	0.97 [0.57–1.62] 1.07 [0.51–2.23]	0.90 0.87			0
Treatment center (compared to Center 1) Center 2 Center 3 Center 4 Center 5	1.12 [0.51–2.49] 1.06 [0.54–2.09] 0.92 [0.41–2.09] 0.69 [0.32–1.51]	0.77 0.87 0.85 0.36			0
**Former RT to the prostatic bed**	**1.56 [1.01–2.41]**	**0.046**	0.78 [0.46–1.34]	0.37	0
Biologically equivalent dose (BED)	1.002 [0.998–1.005]	0.33			

HR, hazard ratio; Obs., observations; ADT, androgen deprivation therapy; PSA, prostate serum antigen; RT, radiotherapy; SBRT, stereotactic body radiotherapy; WPRT, whole-pelvic radiotherapy.Bold values indicate factors with a statistically significant association with FFS.

ADT effect was independent of the treatment, as it increased FFS in both groups (data not shown), but ADT did not impact OS (**Supplementary Figure S1**). In patients who did not receive concurrent ADT, FFS remained significantly shorter in the SBRT population (p = 0.0055), and OS was significantly longer in the SBRT population than in the whole-pelvic IMRT population (p = 0.041) (**Supplementary Figure S2**). In our analysis of factors associated with distant progression (outside the treated nodal area), we found that ADT was the only factor that remained statistically significant in association with distant progression (**Supplementary Table S7**).

In the SBRT group, univariable analyses showed that factors associated with a longer FFS were associated with ADT at recurrence, lower PSA at recurrence, and PSA doubling time >6 months at recurrence. All those factors remained predictive of a longer FFS in the multivariable Cox model along with initial Gleason <8 (**Table 4**).

**Table 4:** Univariable and multivariable analyses of FFS (Cox survival model) in the nodal SBRT-treated population.

Parameter	Univariable HR	p-Value	Multivariable HR	p-Value	Missing Obs.
**Use of ADT at recurrence**	**0.39 (0.18–0.83]**	**0.02**	**0.25 [0.10–0.68]**	**<0.01**	**0**
PSA at diagnosis	0.99 [0.97–1.01]	0.56			15
**Gleason ≥ 8**	1.88 [0.90–3.90]	0.09	**2.73 [1.17–6.4]**	**0.02**	**3**
Interval to biological failure	0.95 [0.88–1.03]	0.23			0
**PSA at recurrence**	**1.13 [1.03–1.24]**	**0.01**	**1.17 [1.04–1.32]**	**<0.01**	**0**
PSA doubling time at Failure (linear)	0.97 [0.93–1.01]	0.19			
**PSA doubling time at failure >6 months**	**0.56 [0.32–0.98]**	**0.04**	**0.44 [0.23–1.01]**	**0.02**	**7**
Iliac common LN	1.79 [0.89–3.60]	0.097			**0**
Number of treated LN (compared to 1) 2 3 to 5	1.16 [0.58–2.34] 1.21 [0.16–8.88]	0.67 0.85			0
Dose ≥ 36 Gy	1.26 [0.73–2.19]	0.4			0
LINAC-delivered SBRT (compared to CyberKnife)	0.55 [0.30–1]	0.05	0.58 [0.26–1.29]	0.18	3
Former RT to the prostatic bed	0.64 [0.35–1.17]	0.15	0.48 [0.23–1.00]	0.05	0

HR, hazard ratio; Obs., observations; ADT, androgen deprivation therapy; PSA, prostate serum antigen; RT, radiotherapy; LN, lymph node; LINAC, linear accelerator.Bold values indicate factors with a statistically significant association with FFS.

## Discussion

In this multicentric retrospective cohort study, we confirmed that the main predictive factors for nodal treatment outcome were RT technique and associated ADT. However, whereas SBRT treatment was associated with a shorter FFS than whole-pelvic IMRT as in former studies, it left further salvage options available and presented a better toxicity profile. For the WPRT treatment, we also observed that the SBRT boost was associated with a worse FFS than the IMRT boost. SBRT was often performed without ADT, after prior prostate bed RT, and chosen based on dosimetric analysis or patient preferences. Those nodes were thus largely in formerly irradiated areas. Upon failure, it was associated with a higher rate of nodal re-irradiation. Factors associated with better SBRT FFS included concomitant ADT, lower PSA at recurrence, PSA doubling time >6 months, and Gleason < 8. Thus, while WPRT remains the reference for nodal recurrence treatment, SBRT remains a good option for properly selected patients.

Associated ADT was an independent factor for good FFS, as it was associated with a better FFS in both treatment groups. It was the only factor associated with less distant progressions, but we have to note that separating local from distant progressions lowers the number of observed events and the probability of showing a difference between groups. It was not associated with a better OS in our cohort, as in former studies (24). Better OS for SBRT treatment over WPRT in ADT-negative patients probably indicates a selection of slowly evolutive patients for this treatment strategy. However, at failure for nodal recurrence treatment, all ADT-negative patients began ADT. It raises thus the question of whether RT performed without ADT can modulate hormone sensitivity of cancer cells at failure depending on the RT modality chosen. We also have to note that the follow-up of 68 months of our study can make conclusions about overall survival poorly reliable.

In the WPRT group, the better performance of IMRT over SBRT boost may be due to different margin definitions around the involved node and deserves further investigation. It is to be noted that different treatment modalities were employed in different centers, but the treatment center was not associated with different outcomes, likely because each modality was used by several centers. Studies regarding repositioning during treatment and further attention to the new MRI-LINAC modalities (29) are also warranted by this work.

In this study, we confirm the better FFS of whole-pelvic IMRT over nodal SBRT observed in previous monocentric and multicentric cohorts (14, 15) and in agreement with the preliminary results of PEACE V-STORM trial presented at the ESTRO 2024 congress (31). This trial demonstrates a disease control advantage from WPRT, comparable acute toxicities, similar quality of life outcomes (27), and identical 2-year toxicity profiles as presented at the European Association of Urology (EAU) 2024 congress (19), establishing WPRT as a standard of care for patients eligible for treatment. However, our study outlines the clinical scenarios in which SBRT is typically used, particularly in patients who are unable to undergo WPRT due to dosimetric constraints. Also, predictive factors for SBRT efficacy may help to avoid SBRT use without ADT in patients who are less likely to benefit long-term from this approach. Prospective trial results are awaited.

We have to note that the preferential use of SBRT in patients previously irradiated on the prostate bed for a biochemical recurrence may have contributed to the worse outcome of the SBRT group. Previous irradiation to the prostatic bed was associated with an increased risk of recurrence in univariable analyses; however, this was no longer significant in multivariable analyses when accounting for the irradiation technique (**Table 3**). This suggests that the preferential use of SBRT in men who had previously undergone prostatic bed irradiation for biochemical recurrence resulted in poorer outcomes. It may indicate that a subset of these patients received suboptimal treatment during their first biochemical recurrence, as many in the cohort underwent prostatic bed irradiation without the benefit of metabolic imaging at the time of recurrence after prostatectomy.

Imaging modality plays a significant role in this study. We observed that PSA levels at diagnosis were elevated, even in patients experiencing their first recurrence after prostate surgery, likely due to the predominant use of PET choline for metabolic imaging during the inclusion period. Despite imaging being performed once PSA levels had risen significantly, some pathological lymph nodes may have gone undiagnosed. Given that PET prostate-specific membrane antigen (PSMA) has a higher detection rate, especially at lower PSA values (30), its routine use could potentially improve the outcomes of nodal SBRT.

Some factors formerly associated with recurrence treatment outcomes were predictive of a better FFS with SBRT only and not in the whole cohort, such as PSA doubling time or Gleason ≥ 8. Other formerly reported predictive factors for nodal recurrences, such as IBF (15), were not identified in our work. The exclusion of lumboaortic recurrences due to their metastatic qualification in the TNM staging system, along with other more stringent inclusion factors such as the exclusion of partial pelvic IMRT fields and the large use of PET choline for node identification, may explain those differences. However, lumboaortic recurrences warrant particular attention, as they were the primary site of recurrence in the OLIGOPELVIS phase II trial (30). These cases may especially benefit from treatment intensification strategies, as explored in the CARLHA phase II trial (31). Corresponding phase III trials are currently ongoing. Also, we did not use a prospective dataset to assess predictive factors for treatment outcomes. However, we could confirm that predictive factors identified for SBRT treatment were specific, as they did not hold true in the whole population (**Table 3**) and the whole pelvis-treated patients (data not shown).

Our study was conducted across five treatment centers and included 64 patients treated by SBRT following a homogeneous initial treatment trajectory. To our knowledge, this is the only retrospective study that outlines predictive factors associated with SBRT outcomes in a multicentric analysis. However, it remains a retrospective work, and the stringency of inclusion factors implied strong patient selection. Each hospital performed approximately four times more nodal recurrence treatments, indicating that SBRT is performed in many other clinical situations. Also, we cannot exclude some additional predictive factors missed by our analysis.

In conclusion, our study confirms better FFS for WPRT but shows that SBRT may be used in some prostate cancer nodal recurrences, particularly in patients with initial Gleason < 8, low PSA at recurrence, and long PSA doubling time at recurrence. The better outcome of IMRT over SBRT boost suggests a need for a more in-depth investigation of margins used for planning.

## Data Availability

The raw data supporting the conclusions of this article will be made available by the authors, without undue reservation.
