# Supplementary figures and images for: Nodal radiotherapy for prostate adenocarcinoma recurrence: predictive factors for efficacy
Source: Front Oncol. 2024 Oct 25;14:1468248. doi: 10.3389/fonc.2024.1468248 (PMC11543566; doi:10.3389/fonc.2024.1468248)

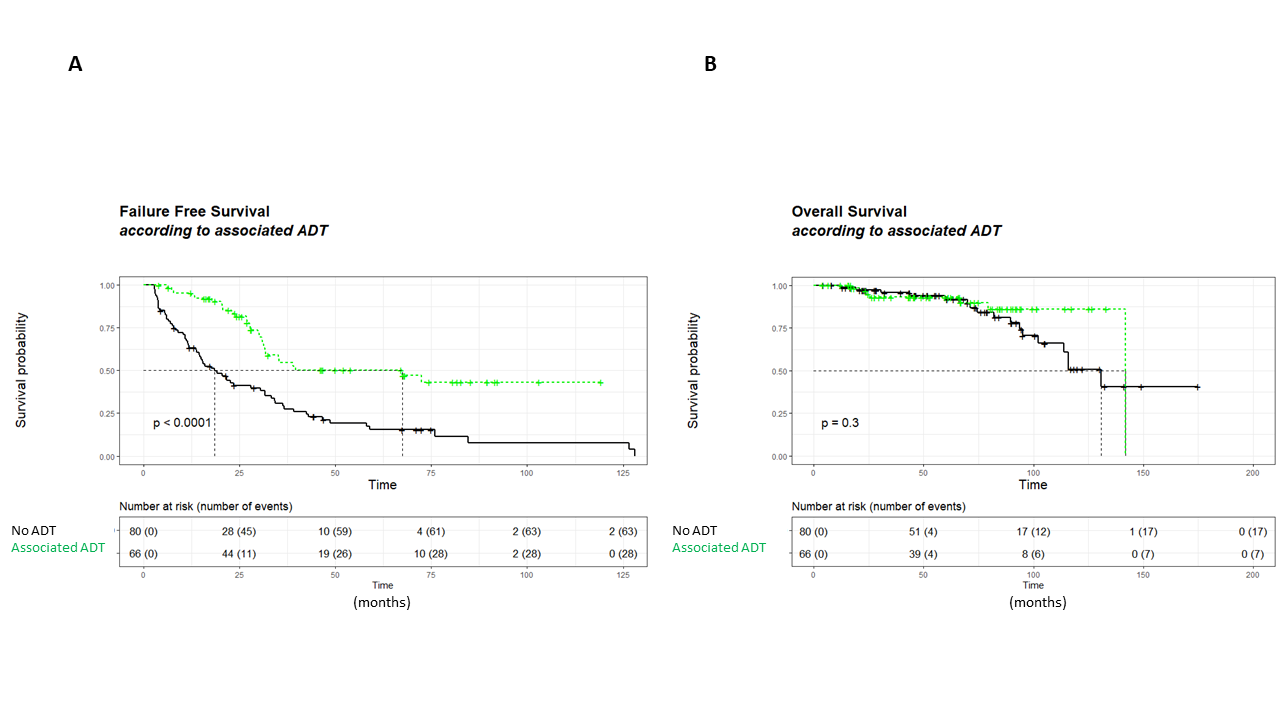

Supplement: Supplementary file 1 [file Image1.tif]

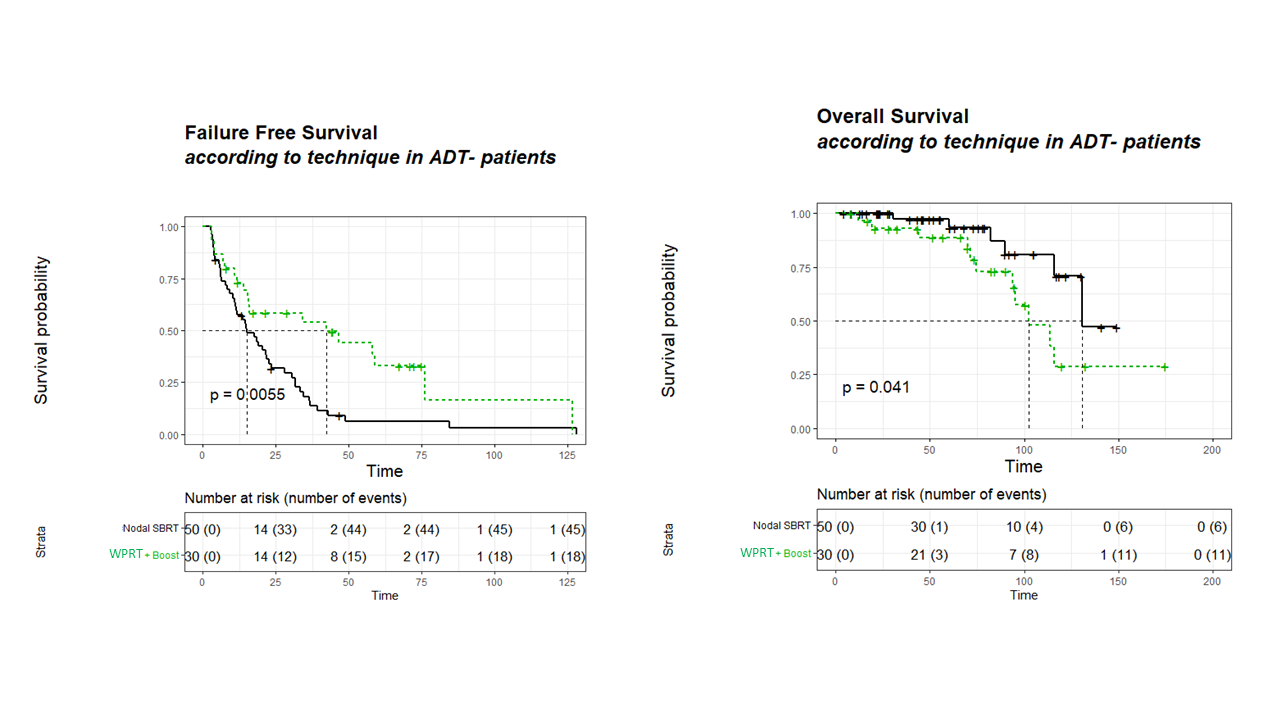

Supplement: Supplementary file 2 [file Image2.tif]
