# Supplementary material for: Nodal radiotherapy for prostate adenocarcinoma recurrence: predictive factors for efficacy
Source: Front Oncol. 2024 Oct 25;14:1468248. doi: 10.3389/fonc.2024.1468248 (PMC11543566; doi:10.3389/fonc.2024.1468248)
Supplement: Supplementary file 3 [file Table1.docx]

|  | Total (N=147) | Nodal SBRT (N=64) | WPRT + Boost (N=83) | p value |
| --- | --- | --- | --- | --- |
| Age at Diagnosis (years) |  |  |  | 0.12 (1) |
| - Median (Range) | 63.1 (45.7, 79.7) | 62.4 (45.7, 78.0) | 63.3 (48.3, 79.7) |  |
| Age at first Nodal recurrence Radiotherapy (years) |  |  |  | 0.30 (1) |
| - Median (Range) | 69.9 (53.3, 88.0) | 69.5 (53.4, 88.0) | 70.8 (53.3, 86.4) |  |
| Treatment Center |  |  |  | 0.33 (2) |
| - 1 | 24 (16%) | 6 (9%) | 18 (22%) |  |
| - 2 | 24 (16%) | 14 (22%) | 10 (12%) |  |
| - 3 | 51 (35%) | 21 (33%) | 30 (36%) |  |
| - 4 | 21 (14%) | 10 (16%) | 11 (13%) |  |
| - 5 | 27 (18%) | 13 (20%) | 14 (17%) |  |
| Type of medical imaging performed for diagnosis |  |  |  | 0.45 (3) |
| - IRM | 3 (2%) | 0 (0%) | 3 (4%) |  |
| - PET choline | 133 (90%) | 58 (91%) | 75 (90%) |  |
| - PET fluciclovine | 2 (1%) | 1 (2%) | 1 (1%) |  |
| - PET PSMA | 9 (6%) | 5 (8%) | 4 (5%) |  |

1. Wilcoxon rank sum test; 2. Pearson’s Chi-squared test; 3. Fisher’s Exact Test for Count Data, SBRT: Stereotaxic Body Radiation Therapy, WPRT: Whole Pelvic Radiation Therapy
