# Supplementary material for: Nodal radiotherapy for prostate adenocarcinoma recurrence: predictive factors for efficacy
Source: Front Oncol. 2024 Oct 25;14:1468248. doi: 10.3389/fonc.2024.1468248 (PMC11543566; doi:10.3389/fonc.2024.1468248)
Supplement: Supplementary file 4 [file Table2.docx]

|  | Total | | | Nodal SBRT | | | WPRT + Boost | | |
| --- | --- | --- | --- | --- | --- | --- | --- | --- | --- |
| Initial treatment | Surgery | Surgery and Prostate Bed radiotherapy | Other | Surgery | Surgery and Prostate Bed radiotherapy | Other | Surgery | Surgery and Prostate Bed radiotherapy | Other |
| number of patients | 54 | 74 | 19 | 14 | 42 | 8 | 40 | 32 | 11 |
| PSA value at nodal recurrence -median (min - max) | 1.94 (0.11-20) | 1.99  (0.14-11.6) | 4.4  (1.2-15) | 1.72  (0.11-8.38) | 1.77  (0.14-11.6) | 2.99  (1.2-13.8) | 2  (0.28-20) | 2.2  (0.88-4.76) | 5.4  (1.53-15) |

n= number of patients treated with this regimen, min = minimum, max=maximum

*One patient with non-reported initial treatment is missing from this table
