# Supplementary material for: Nodal radiotherapy for prostate adenocarcinoma recurrence: predictive factors for efficacy
Source: Front Oncol. 2024 Oct 25;14:1468248. doi: 10.3389/fonc.2024.1468248 (PMC11543566; doi:10.3389/fonc.2024.1468248)
Supplement: Supplementary file 5 [file Table3.docx]

| Reason for SBRT choice |  |  |
| --- | --- | --- |
| - Dosimetric analysis |  | 18 (67%) |
| - Former toxicity of RT |  | 3 (11%) |
| - Medical history |  | 2 (7%) |
| - Patient choice |  | 4 (15%) |
| - Missing |  | 37 |
