# Supplementary material for: Nodal radiotherapy for prostate adenocarcinoma recurrence: predictive factors for efficacy
Source: Front Oncol. 2024 Oct 25;14:1468248. doi: 10.3389/fonc.2024.1468248 (PMC11543566; doi:10.3389/fonc.2024.1468248)
Supplement: Supplementary file 6 [file Table4.docx]

|  | Total | Nodal SBRT | WPRT + Boost | |
| --- | --- | --- | --- | --- |
|  |  |  | IMRT boost | SBRT boost |
| most frequent dose regimens (n) |  | 6 x 6 Gy (17) | 25 x 2 Gy + 14 Gy in 2.3 Gy/fraction SIB (10) | 23 x 2 Gy + 3 x 6 Gy SBRT boost (14) |
|  |  | 5 x 7 Gy (13) | 27 x 2 Gy + 12 Gy in 2.2 Gy/fraction SIB (12) | 23 x 2 Gy + 6 x 6 Gy SBRT boost (4) |
|  |  | 3 x 9 Gy (8) | 25 x 2 Gy + 8 x 2 Gy sequential boost (4) | 23 x 2 Gy + 5 x 7 Gy SBRT boost (1) |
| other regimen (n) |  | (26) | (33) | (5) |
| mean BED (IQR)* | 170 (46.6) | 189 (18.3) | 154 (51.4) | |
|  |  |  | 153 (11.6) | 197 (90) |

n= number of patients treated with this regimen, SIB: Simultaneous Integrated Boost

BED= Biologically Equivalent Dose, * calculated for the dose delivered to the positive nodes with an α/β ratio of 1.5, IQR= Inter-Quartile Range
