# Supplementary material for: Nodal radiotherapy for prostate adenocarcinoma recurrence: predictive factors for efficacy
Source: Front Oncol. 2024 Oct 25;14:1468248. doi: 10.3389/fonc.2024.1468248 (PMC11543566; doi:10.3389/fonc.2024.1468248)
Supplement: Supplementary file 7 [file Table5.docx]

|  | Total (N=147) | Nodal SBRT (N=64) | WPRT + Boost (N=83) | p value |
| --- | --- | --- | --- | --- |
| Acute Urological Toxicity Grade >=II | 15 (10%) | 2 (3%) | 13 (16%) | 0.01 (2) |
| Acute Digestive Toxicity Grade >= II | 4 (3%) | 0 (0%) | 4 (5%) | 0.13 (2) |
| Late Urological Toxicity Grade >=II | 28 (19%) | 6 (9%) | 22 (27%) | < 0.01 (1) |
| Late Digestive Toxicity Grade >=II | 12 (8%) | 1 (2%) | 11 (13%) | 0.01 (2) |
| Acute or late grade III toxicity | 10 (7%) | 2 (3%) | 8 (10%) | 0.19 (2) |

1. Pearson’s Chi-squared test 2. Fisher’s Exact Test for Count Data, SBRT: Stereotaxic Body Radiation Therapy, WPRT: Whole Pelvic Radiation Therapy, PSA: Prostate Serum Antigen
