# Supplementary material for: Nodal radiotherapy for prostate adenocarcinoma recurrence: predictive factors for efficacy
Source: Front Oncol. 2024 Oct 25;14:1468248. doi: 10.3389/fonc.2024.1468248 (PMC11543566; doi:10.3389/fonc.2024.1468248)
Supplement: Supplementary file 8 [file Table6.doc]

|  |  |  | WPRT + Nodal IMRT (N=59) | WPRT + Nodal SBRT (N=24) |  |  |
| --- | --- | --- | --- | --- | --- | --- |
| Acute Urological Toxicity Grade >=II |  |  | 12 (20%) | 1 (4%) |  |  |
| Acute Digestive Toxicity Grade >= II |  |  | 2 (3%) | 2 (8%) |  |  |
| Late Urological Toxicity Grade >=II |  |  | 16 (27%) | 7 (29%) |  |  |
| Late Digestive Toxicity Grade >=II |  |  | 9 (15%) | 2 (8%) |  |  |
| Acute or late grade III toxicity |  |  | 6 (10%) | 2 (8%) |  |  |

Fisher’s Exact Test for Count Data, SBRT: Stereotaxic Body Radiation Therapy, WPRT: Whole Pelvic Radiation Therapy
