# Supplementary material for: Nodal radiotherapy for prostate adenocarcinoma recurrence: predictive factors for efficacy
Source: Front Oncol. 2024 Oct 25;14:1468248. doi: 10.3389/fonc.2024.1468248 (PMC11543566; doi:10.3389/fonc.2024.1468248)
Supplement: Supplementary file 9 [file Table7.docx]

**Univariable and Multivariable analyses of Distant progression (cox survival model) in the whole population**

| Variable | Univariable HR | p val | Multivariable HR | p val | Missing Obs. |
| --- | --- | --- | --- | --- | --- |
| **Use of ADT at recurrence** | **0.40 [0.24 – 0.65]** | **<0.001** | **0.45 [0.24 – 0.85]** | **0.01** | **1** |
| PSA doubling time (linear) | 0.99 [0.95 – 1.02] | 0.40 |  |  | 21 |
| PSA doubling time > 6 month | 0.90 [0.56 – 1.44] | 0.66 |  |  | 21 |
| Time from diagnosis | 0.99 [0.94 – 1.05] | 0.82 |  |  | 0 |
| PSA at diagnosis | 1.01 [0.99 – 1.03] | 0.25 | 1.00 [0.99 – 1.02] | 0.72 | 30 |
| PSA at recurrence | 1.03 [0.91 – 1.04] | 0.38 |  |  | 0 |
| Gleason ≥8 at diagnosis | 0.65 [0.33 – 1.27] | 0.21 | 0.77 [0.37 – 1.59] | 0.48 | 5 |
| **Treatment modality (compared to SBRT)**  **- WPRT + Nodal IMRT**  - WPRT + Nodal SBRT | **0.47 [0.29 – 0.76]**  0.61 [0.29 – 1.31] | **<0.001**  0.20 | 0.82 [0.45 – 1.49]  0.89 [0.34 – 2.37] | 0.52  0.81 | 0 |
| Number of treated LN (compared to 1)  2  3 to 5 | 1.12 [0.65 – 1.95]  1.46 [0.69 – 3.09] | 0.67  0.32 |  |  | 0 |
| Treating Center (Compared to Centre 1)  Centre 2  Centre 3  Centre 4  Centre 5 | 1.14 [0.43 – 3.09]  1.76 [0.78 – 3.98]  1.29 [0.49 – 3.40]  0.79 [0.31 – 1.97] | 0.79  0.17 0.61  0.61 |  |  | 0 |
| **Former RT to the prostatic bed** | **2.22 [1.35 – 3.65]** | **0.002** | 1.52 [0.86 – 2.66] | 0.14 | 0 |
| Biologically Equivalent Dose (BED) | 1.002 [0.998 – 1.005] | 0.28 |  |  |  |

HR: Hazard Ratio, Obs.: Observations, ADT: Androgen Deprivation Therapy, PSA: Prostate Serum Antigen, RT: Radiation Therapy, SBRT: Stereotaxic Body Radiation Therapy, WPRT: Whole Pelvic Radiation Therapy
